# Neurological soft signs in Tunisian patients with first-episode psychosis and relation with cannabis use

**DOI:** 10.1186/s12991-017-0153-3

**Published:** 2017-07-12

**Authors:** Ahmed Mhalla, Bochra Ben Mohamed, Christoph U. Correll, Badii Amamou, Anouar Mechri, Lotfi Gaha

**Affiliations:** 1 Psychiatry Department, Fattouma Bourguiba Hospital, 5000 Monastir, Tunisia; 2 Faculty of Medicine of Monastir, University of Monastir, Monastir, Tunisia; 3 Department of Psychiatry, The Zucker Hillside Hospital, Glen Oaks, NY USA; 4 Department of Psychiatry and Molecular Medicine, Hofstra Northwell School of Medicine, Hempstead, NY USA; 5 Center for Psychiatric Neuroscience, Feinstein Institute for Medical Research, Manhasset, NY USA

**Keywords:** Schizophrenia, First episode, Cannabis, Neurological soft signs

## Abstract

**Background:**

Neurological soft signs (NSS) are minor non-localizing neurological abnormalities that are conceptualized as neurodevelopmental markers that mediate the biological risk for psychosis. We aimed to explore the relationship between NSS and cannabis use, an environmental risk factor of psychosis.

**Methods:**

This was a cross-sectional study in consecutively admitted patients hospitalized for first-episode psychosis. NSS were assessed by the NSS scale (23 items exploring motor coordination, motor integrative function, sensory integration, involuntary movements or posture, quality of lateralization). Presence of NSS was defined as a NSS scale total score ≥9.5. Cannabis use was ascertained with the cannabis subsection in the Composite International Diagnostic Interview.

**Results:**

Among 61 first-episode psychosis patients (mean age = 28.9 ± 9.4 years; male = 86.9%, antipsychotic-naïve = 75.4%), the prevalence of current cannabis use was 14.8% (heavy use = 8.2%, occasional use = 6.6%). NSS were present in 83.6% of the sample (cannabis users = 66.7% versus cannabis non-users = 85.5%, *p* = 0.16). The mean total NSS score was 15.3 ± 6.7, with a significant lower total NSS score in cannabis users (11.2 ± 5.6 versus 16.0 ± 6.7, *p* = 0.048). Differences were strongest for the “motor coordination” (*p* = 0.06) and “involuntary movements” (*p* = 0.07) sub-scores.

**Conclusions:**

This study demonstrated a negative association between cannabis use and NSS, especially regarding motor discoordination. This finding supports the hypothesis that a strong environmental risk factor, such as cannabis, may contribute to the onset of psychosis even in the presence of lower biological and genetic vulnerability, as reflected indirectly by lower NSS scores. Nevertheless, additional studies are needed that explore this interaction further in larger samples and considering additional neurobiological and environmental risk factors.

## Introduction

Neurological soft signs (NSS) are minor non-localizing neurological abnormalities determined by clinical examination [1]. NSS concern four main areas of neurological functioning: motor coordination, sensory integration, sequencing of complex motor acts, and primitive developmental reflexes [2]. NSS have been conceptualized as neurodevelopmental markers that mediate the biological propensity for the development of psychosis. This conceptualization was established on the basis of many observations showing higher rates of NSS not only among people with schizophrenia, but also among treatment-naive patients with first-episode psychosis (FEP) [3, 4], non-psychotic siblings, and subjects considered at high risk for psychosis [5–8]. The prevalence of NSS in patients with FEP has been reported to range from 20 to 97%, depending on the study sample and methodology, and NSS seem to precede psychotic symptoms [3, 4, 9].

Cannabis is the most widely used illicit drug in patients with psychosis [10], and the median cannabis use disorder rates are as high as 28.6% for current and 44.4% for life-time prevalence [11]. Longitudinal studies have reported an increased likelihood for developing schizophrenia and other psychoses after cannabis use [12–17], especially when cannabis use has been moderate to severe and/or started in the early teens [14, 18–20]. The relationship between cannabis and psychosis seems fairly specific to schizophrenia, as compared to other mental disorders [21, 22]. This relationship cannot be explained by potentially confounding factors, such as premorbid disorders, other types of drug use, intoxication effects, personality traits, sociodemographic markers, or intellectual ability [22]. Accordingly, several reviews conclude that there is an increased risk for psychosis in individuals who have used cannabis, typically in the magnitude of an odds ratio of 1.5–2 [22–24]. However, there are also opposing views on cannabis as a risk factor for psychosis. Some authors propose that there is a causal relationship between cannabis use and psychosis [25, 26]. Others suggest that cannabis use only precipitates psychosis in vulnerable individuals on their pathway to the disorder [25–27]. Cannabis consumption usually precedes the onset of psychosis [28, 29]. However, most individuals do not develop psychosis after cannabis use, suggesting that risk of psychosis must be modulated by other factors. In line with this conceptualization, data from recent comprehensive studies suggest that cannabis is an environmental risk factor that interacts with genetic and biological vulnerabilities for psychosis [30, 31].

While different authors have studied the association between NSS and perinatal factors, such as obstetric complications [32–34], few studies have investigated the interaction between NSS and non-perinatal environmental factors, such as cannabis use [35–37].

The aim of this study was to explore the relationship between neurodevelopmental markers reflecting neurobiological vulnerability (NSS) and an environmental risk factor (cannabis use) in a sample of Tunisian patients with FEP. The hypothesis was that the cannabis pathway to psychosis may reflect less neurobiological vulnerability.

## Patients and methods

### Study design

This was a cross-sectional study conducted over a period of 14 months (from July 2012 to September 2013) in the psychiatry department of Fattouma Bourguiba Hospital in Monastir, Tunisia, in consecutively admitted patients hospitalized for FEP according to Diagnostic and Statistical Manual of Mental Disorders (DSM-IV) criteria [38]. Patients had the diagnosis of schizophrenia, schizophreniform disorder, brief psychotic disorder, delusional disorder, substance-induced psychotic disorder, or psychotic disorder not otherwise specified. Exclusion criteria were: age >55 years old, prior hospitalization or consultation in a psychiatric unit, diagnosis of psychotic disorder due to medical condition, mental retardation, a history of major neurological disorder and unwillingness to consent to participate in the study.

### Measures and assessment tools

Sociodemographic and clinical data were collected both with a pre-established questionnaire and based on medical record review. The premorbid functioning was evaluated by the Premorbid Adjustment Scale (PAS) [39, 40] based on patient interview, the duration of untreated psychosis (DUP) was estimated by interviewing the caregiver/family and the patient, the psychometric assessment was conducted by the Positive and Negative Syndrome Scale (PANSS) [41], and the Global Assessment of Functioning scale (GAF) [42] based on patient interview.

The neurological evaluations were carried-out using the neurological soft signs (NSS) scale by Krebs et al. The NSS scale explores 23 minor neurological signs that are rated from 0 to 3 and distributed in five main domains: motor coordination, motor integration, sensory integration, involuntary movements or posture, and quality of lateralization. The threshold value for this scale was fixed at 9.5 as recommended in the original version [43]. Neurological side effects of antipsychotics were evaluated by the Simpson Angus (SA) scale [44].

The PANSS and the GAF scales were administered within 72 h of the patient’s hospitalization. The NSS scale and the Simpson Angus scale were completed within seven days of hospitalization.

We ascertained the use of cannabis with the cannabis subsection of the Composite International Diagnostic Interview (CIDI), included within the section of substance use. According to the CIDI, patients were considered to be cannabis users if they had taken cannabis on five or more occasions; patients were considered as “heavy cannabis users” when the frequency of cannabis use was daily or nearly every day.

### Statistical analyses

All statistical analyses were performed with SPSS for Windows, Version 21.0.

The independent factor was cannabis use, which divided the study sample in two groups: in-patients with current cannabis use versus in-patients without current cannabis use. The Mann–Whitney non-parametric test, the Chi-square test, the Fisher’s exact test and the Pearson correlation coefficient were used for the between-group analysis. The statistical significance was set at 5%. Additionally, for the presence/absence of NSS, defined by the threshold value of >9.5 on the NSS Scale, we performed a logistic regression with cannabis use as well as smoking, alcohol use, PANSS positive score, PANSS negative score, PANSS disorganization score, PAS total score, and Simpson Angus score as variables entered into the model. The variables included were the significant ones at the statistical threshold of 0.25. All tests were two-sided with *α* = 0.05. Due to the exploratory nature of the analyses, we did not correct for multiple comparisons.

## Results

### Sociodemographic, clinical, therapeutic use characteristics

At the end of the study period, 71 consecutively enrolled patients met the inclusion and exclusion criteria. Of these, 10 were not recruited: 4 patients due to premature discharge against medical advice and 6 patients refused study participation. Altogether, 61 in-patients were included in this study.

The study sample contained 53 men (86.9%) and 8 women; the mean age was 28.9 ± 9.4 year-old. The majority had a low educational level or was unschooled (70.5%) and single (75.4%). Family history of mental illness was present in 24.6% of the patients; consisting mainly of psychotic disorders in first-degree relatives (Table 1). The majority of the patients (67.2%) had never taken psychotropic treatment before the hospitalization; only 24.6% had received antipsychotic treatment, most often only for a few days before hospitalization and 8.2% had received antidepressant treatment. The main diagnosis was schizophreniform disorder (42.6%), the mean DUP was 39.6 ± 63.7 weeks; and the majority of patients were treated with first-generation antipsychotics (68.8%) (Table 2).

**Table 1:** Sociodemographic characteristics

Variables	Total number of patients (*n* = 61)	Cannabis users (*n* = 9)	Cannabis non-users (*n* = 52)	*p*
Age	28.9 ± 9.4	27.4 ± 7.8	29.2 ± 10	0.81
Male sex	53 (86.9%)	9 (100%)	44 (84.6%)	0.61
Psychiatric family history	15 (24.6%)	2 (22.2%)	13 (25.0%)	0.33
University studies	18 (29.5%)	3 (33.3%)	15 (28.8%)	0.57
Marital status				
Single	46 (75.4%)	7 (77.8%)	39 (75%)	0.72
Married	11 (18%)	1 (11.1%)	10 (19.2%)	
Divorced	4 (6.6%)	1 (11.1%)	3 (5.8%)	
Currently employed	27 (44.3%)	8 (88.9%)	6 (11.5%)	0.91
Premorbid Adjustment Scale score	19.2 ± 7.9	18.7 ± 8.5	22.6 ± 7.6	0.23

**Table 2:** Clinical and therapeutic characteristics

Variables	Total number of patients (*n* = 61)	Cannabis users (*n* = 9)	Cannabis non-users (*n* = 52)	*p*
Psychiatric diagnosis				
Schizophrenia	20 (32.9%)	5 (55.5%)	15 (28.8%)	
Schizophreniform disorder	26 (42.6%)	1 (11.1%)	25 (48.1%)	
Brief psychotic disorder	10 (16.4%)	1 (11.1%)	9 (17.2%)	0.10
Cannabis-induced psychosis	3 (4.9%)	2 (22.2%)	1 (1.9%)	
Delusional psychosis	1 (1.6%)	0 (0%)	1 (1.9%)	
Psychotic disorder not otherwise specified	1 (1.6%)	0 (0%)	1 (1.9%)	
PANSS total score	99.5 ± 20.6	101.6 ± 20.5	99.1 ± 20.8	0.65
PANSS positive score	25.1 ± 6	27 ± 6.3	24.8 ± 6	0.43
PANSS negative score	24.3 ± 9.3	25.7 ± 9.8	24 ± 9.3	0.56
GAF score	32.4 ± 8	30 ± 5.6	33.5 ± 8.3	0.28
Duration of untreated psychosis (weeks)	39.6 ± 63.7	28.6 ± 35.2	41.5 ± 87.4	0.45
Antipsychotic treatment				
First-generationantipsychotic	25(41%)	4 (44.4%)	21 (40.4%)	
Second-generationantipsychotic	19 (31.1%)	2 (22.2%)	17 (32.7%)	0.84
Co-treatment of first- and second-generation antipsychotics	17 (27.8%)	3 (33.3%)	14 (26.9%)	
Chlorpromazine equivalent	619.8 ± 419.8 mg	914.0 ± 629.8 mg	568.8 ± 356.6 mg	0.16
Simpson Angus scale score	3.8 ± 3.2	4.3 ± 2.6	3.7 ± 3.	0.37
Duration of hospitalization (days)	29.1 ± 16.6	43.3 ± 15.5	26.7 ± 15.6	0.005

*GAF* Global Assessment of Functioning, *PANSS* Positive and Negative Symptom Scale

### Cannabis use

The prevalence of the current cannabis use in this population was 14.8%, with heavy use among 8.2% of the patients and occasional use among 6.6%.

### Neurological soft signs (NSS)

#### NSS evaluation

The mean NSS score was 15.3 ± 6.7 (ranging from 4 to 32.5). The highest sub-scores were noted in the domain of motor coordination (6.1 ± 2.7) (Table 3). Using the threshold value of ≥9.5 on the NSS scale, NSS were present in 83.6% of the total patient sample.

**Table 3:** Correlations between NSS and clinical and therapeutic characteristics

	NSS total score
PAS total score	*r* = 0.32, *p* = 0.04
PANSS total score	*r* = 0.36, *p* = 0.005
PANSS positive score	r = −0.06, p = 0.69
PANSS negative score	*r* = 0.45, *p* < 0.001
PANSS disorganization score	*r* = 0.41, *p* = 0.001
CGI-severity score	*r* = 0.30, *p* = 0.02
GAF score	*r* =−0.26, *p* = 0.04
SA score	*r* = 0.52, *p* < 0.001

*NSS* neurological soft signs, *PAS* Premorbid Adjustment Scale, *PANSS* Positive and Negative Syndrome Scale, *GAF* Global Assessment of Functioning, *CGI* Clinical Global Impression, *SA* Simpson Angus

#### NSS and clinical and therapeutic characteristics

Correlations were found between the NSS total scores and the Poor Premorbid Functioning (*r* = 0.32, *p* = 0.04), the PANSS total scores (*r* = 0.36, *p* = 0.005), and the negative (*r* = 0.45, *p* < 0.001) and disorganization sub-scores (*r* = 0.41, *p* = 0.001), the CGI-severity scores (*r* = 0.30, *p* = 0.02), the impairment functioning in the GAF (*r* = −0.26, *p* = 0.04) and with extrapyramidal symptoms (*r* = 0.52, *p* < 0.001) (Table 3).

#### NSS and cannabis use

Comparing NSS scores between patients with and without cannabis use demonstrated significantly lower total NSS scores of in patients with cannabis use: 11.2 ± 5.6 versus 16.0 ± 6.7 (*p* = 0.048) (Table 4). The linear regression model showed that this association remained significant after adjustment for two potentially confounding factors that have been associated with NSS: negative symptoms and neurological side effects of antipsychotics (Table 5).

**Table 4:** Neurological soft signs scale scores in patients with and without cannabis use

Neurological soft signs scale scores: median, (interquartile range)	Total number of patients (*n* = 61)	Cannabis use		Statistic tests
		Yes (*n* = 9)	No (*n* = 52)	
Motor coordination sub-score	6.0 (4.3, 9.0)	3.5 (2.3,8.0)	6 (4.5, 9.0)	*p* = 0.06
Motor integration sub-score	3.0 (1.5, 5.3)	2.5 (1.3, 4.0)	3(1.5, 5.5)	*p* = 0.51
Sensory integration sub-score	3.5 (1.5, 4.8)	2.0 (1.0, 4.0)	3.5 (1.5, 5.4)	*p* = 0.20
Involuntary movements or posture sub-score	0 (0, 0)	0 (0, 0.3)	0.3 (0, 0.3)	*p* = 0.07
Quality of lateralization sub-score	1.0 (0, 1.0)	1 (0, 1.5)	1.0 (0, 3.0)	*p* = 0.20
Total score	13.5 (11.0, 19.5)	11.5 (6.0, 14.3)	14.5 (11.5, 20.75)	*p* *=* *0.048*
Total score >9.5 (*N*, %)	51 (83.6%)	6 (66.6%)	45 (86.5%)	*p* = 0.16

**Table 5:** Linear regression NSS total scores, PANSS negative scores and Simpson Angus scores

	Standardized coefficient beta	CI	*p* value
Cannabis use	−0.315	(−9.41, −2.41)	0.001
PANSS negative score	0.402	(0.15, 0.42)	<0.001
Simpson Angus score	0.476	(0.59, 1.36)	<0.001

*PANSS* Positive and Negative Symptom Scale

There was also an inverse, but not significant relationship between the use of cannabis and the motor coordination and the involuntary movements sub-scores (Table 3).

There was also a significant association between heavy cannabis use and lower total NSS scores (*p* = 0.048).

Altogether, 66.7% of the patients with cannabis use exceeded the threshold value of 9.5 versus 85.5% of the non-users (*p* = 0.16). Similarly, a logistic regression analysis, with the presence of NSS as dependant variable and cannabis use, smoking, alcohol use, PANSS positive score, PANSS negative score, PANSS disorganization score, PAS total score and Simpson Angus score as covariates, did not show a significant association between presence of NSS and current cannabis use (*p* = 0.12).

## Discussion

In this study, the prevalence of NSS was 83.6%, given the threshold score of 9.5 suggested by the NSS scale authors [43]. Studies that evaluated patients with first-episode psychosis reported a high prevalence of NSS, ranging from 20% for the Scottish Schizophrenia Research Group [9] to 97.1% for Browne et al. [4] (Table 6).

**Table 6:** Prevalence of neurological soft signs in first-episode psychosis

Authors (year)	*N*	Study population	Instrument to assess NSS	Prevalence of NSS (%)
The Scottish Schizophrenia Research Group [9]	49	First-episode schizophrenia	NES scale	20
Flyckt et al. [59]	31	First-episode psychosis	NES scale	78
Browne et al. [4]	35	First-episode schizophrenia or schizophreniform disorder	NES scale CNE scale	97
Emsley et al. [60]	66	First-episode psychosis, schizophreniform disorder or schizoaffective disorder	NES scale	68
Ruiz-Veguilla et al. [36]	92	First-episode psychosis	NES scale	45
Our study	61	First-episode psychosis	NSS scale	84

*CNE* Condensed Neurological Examination, *NES* Neurological Evaluation Scale, *NSS* neurological soft signs, *NSS scale* Neurological Soft Signs scale

In this study, we examined the relationship between cannabis use and NSS in FEP patients; cannabis users had significantly fewer NSS than patients without a history of cannabis use. This findings were similar to those reported by Ruiz-Veguilla et al. [36] who studied cannabis use and NSS among 92 patients with FEP (64% males, mean age: 26.9 ± 10.1 years old). The authors found that heavy cannabis users (55% of the sample of the study) had significantly less NSS assesses with the Neurological Evaluation Scale independent of potential confounders, such as sex, age, family history of psychosis, and negative symptoms [36]. A similar association was also found by Stirling et al. [37] in a sample of 112 non-depressed FEP patients (56.2% males, mean age: 26.3 years old) with 38% of cannabis users. Other studies demonstrated a lower NSS scores for patients with chronic schizophrenia and a history of cannabis use than for those without cannabis use. For example, Bersani et al. [45] investigated NSS in 25 male cannabis-consuming and 25 male non-consuming schizophrenia patients, using the Neurological Evaluation Scale and concluded that non-consuming patients showed a higher incidence of NSS. Joyal et al. [35], in a study of 16 men with and 14 men without a dual diagnosis of drug abuse and schizophrenia, reported that drug abuse was associated to fewer frontal soft signs.

Three possible explanations are suggested for this seemingly paradoxical relationship between cannabis use and NSS in FEP. First, some cannabis components may have neuroprotective effects by inhibiting the glutamatergic excitotoxicity system [46, 47]. Second, this association could be explained by the fact that cannabis would act more directly on the onset of psychosis in genetically less vulnerable individuals [19, 48] since NSS are shown to reflect a genetic liability to psychosis. In this context, cannabis use may be the environmental factor that reveals or potentiates the vulnerability to psychosis. Accordingly, it is likely that cannabis could increase the effects of genetic risk factors for psychosis. Thus, cannabis users may follow a different pathway (with less neurobiological vulnerability factors) in developing psychotic disorders compared to patients without a history of cannabis use [36, 48]. Third, the inverse association between NSS and cannabis use could be explained by a relationship between severe NSS with other clinical characteristics that would limit a subject’s personal access to cannabis [47]. In fact, most studies that examined NSS in FEP, in concordance with the results of this study, concluded that NSS were associated with more negative symptoms [2, 49, 50], disorganization symptoms [2, 49, 50] and illness severity [2]. These illness dimensions can limit the patients’ social interaction abilities and diminish their motivation or ability to obtain cannabis.

This study also showed an inverse, but not significant relationship between the use of cannabis and both “motor coordination” (*p* = 0.06) and “involuntary movements” (*p* = 0.07) sub-scores. To our knowledge, no other study explored the interaction between cannabis use and the different sub-groups of NSS in FEP patients. The available data about this topic are restricted to studies with non-psychotic populations that showed greater impairment of motor functioning in patients with cannabis use. Dervaux et al. [51] compared the impact of cannabis use on NSS among patients with cannabis dependence and healthy controls and demonstrated that higher NSS scores were associated especially with motor coordination difficulties in cannabis users. Roser et al. [52] reported impairment in motor speed after cannabis use. These results could be explained by the important role of the endocannabinoid system in the control of movements. In fact, a prominent distribution of the cannabinoid 1 (CB1) receptors in the basal ganglia has been described in patients with movements disorders [53, 54], and cannabis, when interacting with CB1 receptors, induces dopamine release and an increase in motor response [55]. Heavy cannabis consumption may also lead to deterioration in the control system balance and thereby contribute to motor inhibition [56]. The dose-dependent response of motor coordination to cannabis may be due to the involvement of GABAergic and glutamatergic systems as a target of cannabis and its psychoactive component Delta-(9)-tetrahydrocannabinol (THC) [56], or the development of sensitization and adaptive process, which leads to dopamine decrease in prefrontal regions after repeated use [57, 58]. This finding may explain the impairment of motor skills reported over non-psychotic patients.

Conversely, however, higher cannabis consumption may produce a different response, which consists of a motor stimulation instead of inhibition depending on the adaptive mechanism put in place [47]. It is possible that this stimulatory effect could explain the inverse relation between motor coordination and cannabis in this study. Additionally, we investigated in-patients without any access to cannabis at the time of investigation. Hence, acute cannabis effects would not have been influenced our results and we assessed cannabis use more as a trait marker or risk factor for FEP. The fact that we found less motor coordination in patients with cannabis use and FEP strengthens our hypothesis that cannabis use might bring out psychosis risk in those individuals with less other neurobiological risk factors, as motor dysfunction, together with low intellectual quotient, was identified as one of the two strongest biological markers for schizophrenia risk in a recent meta-analysis [15].

There are several limitations to this study. First, the study was not based on a sample size calculation; the sample size, especially of the cannabis users, is small, although it lies within the range of similar FEP studies on this topic. Second, we did not confirm absence of cannabis use by urine screening and we did not have data regarding the exact time between the NSS evaluation and last cannabis consumption. Third, in view of the need for urgent treatment, it hasn’t been always possible to assess NSS before antipsychotic administration, which would have been better for NSS evaluation. Finally, we did not collect data on the amount of cannabis use.

This study demonstrated a negative association between cannabis use and NSS, especially regarding motor coordination. This finding supports the hypothesis that a strong environmental risk factor, such as cannabis, may contribute to the onset of psychosis even in the presence of lower biological and genetic vulnerability, as reflected indirectly by lower NSS scores. Nevertheless, due to the limitations of our study and its exploratory nature, this question remains open, and additional studies are needed to explore this interaction further. Such studies should have sufficiently large samples of cannabis users and non-users and consider cannabis and NSS in the context of additional neurobiological and environmental risk factors.

## Conclusions

Our study demonstrated a negative association between cannabis use and NSS, especially regarding motor discoordination. This finding supports the hypothesis that a strong environmental risk factor, such as cannabis, may contribute to the onset of psychosis even in the presence of lower biological and genetic vulnerability, as reflected indirectly by lower NSS scores. Nevertheless, additional studies are needed that explore this interaction further in larger samples and considering additional neurobiological and environmental risk factors.

## Abbreviations

NSS: neurological soft signs
FEP: first-episode psychosis
DSM: Diagnostic and Statistical Manual of Mental Disorders
PAS: Premorbid Adjustment Scale
DUP: duration of untreated psychosis
PANSS: Positive and Negative Syndrome Scale
GAF: Global Assessment of Functioning scale
CIDI: Composite International Diagnostic Interview
CB1: cannabinoid 1
THC: Delta-(9)-tetrahydrocannabinol
